# Leucine-Rich Alpha-2 Glycoprotein 1 as a Biomarker for Evaluation of Inflammatory Bowel Disease Activity in Children †

**DOI:** 10.3390/jcm14082803

**Published:** 2025-04-18

**Authors:** Betül Aksoy, Yeliz Çağan Appak, Murat Akşit, Serenay Çetinoğlu, Sinem Kahveci, Şenay Onbaşı Karabağ, Selen Güler, İlksen Demir, İnanç Karakoyun, Maşallah Baran

**Affiliations:** 1 Department of Pediatric Gastroenterology, Hepatology and Nutrition, Faculty of Medicine, Izmir Katip Celebi University, Izmir City Hospital, 35540 Izmir, Turkey; yelizcagan@yahoo.com (Y.Ç.A.); drserenaycetinoglu@gmail.com (S.Ç.); selenguler6@hotmail.com (S.G.); drilksendemir@gmail.com (İ.D.); mbaran2509@gmail.com (M.B.); 2 Department of Medical Biochemistry, Tepecik Training and Research Hospital, 35020 Izmir, Turkey; murataksit3545@gmail.com; 3 Department of Pediatric Gastroenterology, Hepatology and Nutrition, Izmir City Hospital, 35540 Izmir, Turkey; dr_skahveci@hotmail.com (S.K.); senayonbasikarabag@gmail.com (Ş.O.K.); 4 Department of Medical Biochemistry, Izmir City Hospital, 35540 Izmir, Turkey; inanckara70@hotmail.com

**Keywords:** leucine-rich alpha-2 glycoprotein 1, inflammatory bowel disease, biomarker

## Abstract

**Background:** Leucine rich α-2 glycoprotein (LRG) is a glycoprotein that is an acute-phase protein produced by neutrophils, macrophages, hepatocytes, and intestinal epithelial cells. This study aimed to determine the serum LRG (s-LRG) and urine LRG (u-LRG) expression levels in children with inflammatory bowel disease (IBD) and evaluated their correlation with clinical disease activity, other inflammatory markers, laboratory results, and endoscopic activity scoring. **Methods:** This prospective observational study was conducted at a tertiary centre and included children aged 2–18 years with IBD. Clinic activity scoring was used to assess clinical disease activity. Haemoglobin levels, platelet counts, albumin, C-reactive protein, and erythrocyte sedimentation rate were analysed in the blood sample. LRG levels were measured in both blood and urine samples. The endoscopic assessment was scored according to the simple endoscopic score and Mayo endoscopic score. Serum and urine LRG levels were measured using commercial enzyme-linked immunosorbent assay kits. Disease activation was defined based on clinical activity scoring, laboratory results, and endoscopic evaluation. The results were compared between the active IBD and remission groups. **Results:** Forty-two (50%) patients with active IBD and forty-two (50%) patients in remission were included in this study. The serum levels of LRG were elevated in the patients with active IBD compared with the levels in the patients with IBD in remission (*p* = 0.020). However, there was no difference in the u-LRG level between the two groups (*p* = 0.407). In patients with IBD, positive correlations were observed between s-LRG, platelet count, C-reactive protein (CRP), and the erythrocyte sedimentation rate. The serum LRG was negatively correlated with albumin and haemoglobin levels. Urine LRG was not correlated with s-LRG in any patients with IBD included or in patients with active IBD. The cutoff value for s- LRG (77.03 μg/mL) had a sensitivity and specificity of 40.4% (95% CI 25.6–56.7%) and 88.1% (95% CI 74.3–96.0%), respectively. It was found that s-LRG was a more significant parameter than CRP in predicting disease activation. **Conclusions:** This prospective study demonstrated that the s-LRG level is a useful biomarker for predicting disease activation in children with IBD and appears to be a more significant parameter than the CRP level. However, the u-LRG level is not effective in predicting disease activation in children with IBD.

## 1. Background

Inflammatory bowel disease (IBD) is a spectrum of conditions, including Crohn’s disease (CD), ulcerative colitis (UC), and IBD-unclassified (IBD-U), which can be characterised by chronic immune activation and inflammation of the gastrointestinal tract [1]. The incidence of IBD is in the general population is increasing. It is not uncommon in children and up to 25% of patients with IBD are diagnosed before the age of 20 [1]. Environmental and genetic factors, including the microbiome, are involved in the aetiology of IBD, which is known to be multifactorial. Laboratory abnormalities indicative of a chronic inflammatory state, such as anaemia, hypoalbuminemia, and elevated inflammatory markers, are observed in children with IBD. IBD is diagnosed using a combination of clinical, laboratory, imaging and endoscopic parameters, including histopathology [2].

The aim of treating paediatric IBD is to induce and maintain clinical remission, achieve normal growth, provide the best quality of life, promote psychological health, and minimise toxicity as far as possible. Despite treatment, disease activation is frequently observed in children with IBD. Stratifying disease progression and the effects of therapeutic interventions are followed by the use of clinical scoring tools such as the Paediatric Crohn’s Disease Activity Index (PCDAI) and the Paediatric Ulcerative Colitis Activity Index (PUCAI) [3]. The most common way to assess disease activation and mucosal healing is repeated endoscopy with biopsy. However, this technique is invasive, time-consuming, and has an uncomfortable preparatory regimen. Therefore, a non-invasive marker is needed that is accurate, reproducible, standardised, easily interpreted by clinicians, and has high diagnostic sensitivity and specificity correlating with disease activation and mucosal healing. The most widely studied inflammatory markers in IBD are C-reactive protein (CRP) and faecal calprotectin (FC). Although a correlation between endoscopic activity and CRP has been reported, there is insufficient data to support its widespread use in IBD. Faecal calprotectin is very sensitive, but it is not a specific marker for the detection of inflammation in the gastrointestinal tract [1,4]. Moreover, the concentrations of FC in stool samples may differ depending on when they are collected [5]. In addition, it may not be easy to obtain stool samples from children at any time.

Leucine-rich alpha-2 glycoprotein (LRG) is a 50 kDa glycoprotein containing repetitive leucine-rich motif sequences [6]. It was first purified from human serum and has recently been developed as a new serum biomarker [6]. Leucine rich α-2 glycoprotein is an acute-phase protein produced by neutrophils, macrophages, hepatocytes, and intestinal epithelial cells. LRG expression is also induced by other cytokines such as tumour necrosis factor (TNF)-α, IL-1b, and IL-22, unlike CRP, which is induced by interleukin (IL)-6 [7]. Leucine rich α-2 glycoprotein has been linked to various inflammatory states, such as asthma, infections, rheumatoid arthritis, and autoimmune diseases [7]. It has been demonstrated that serum LRG (s-LRG) is a useful biomarker for predicting mucosal inflammation in CD and UC in adult patients with IBD [8]. In addition, serum and urinary LRG (u-LRG) have been shown to be potential non-invasive biomarkers for the diagnosis of acute paediatric appendicitis [9,10]. In children, urine samples are easier to collect than stool samples. There are very few studies of s-LRG in children with IBD for disease activation. This study aimed to determine s-LRG and u-LRG expression levels in children with IBD and evaluate their correlation with clinical disease activity, other inflammatory markers, laboratory results, and endoscopic activity scoring. In addition, this is the first study to assess the utility of u-LRG as an IBD biomarker compared to s-LRG.

## 2. Methods

**Patients:** This prospective observational study was conducted from January to July 2023 in a tertiary centre and with children with IBD who were included in the study. The diagnosis of CD or UC was proven clinically, endoscopically, and histologically. The patients were divided into two groups: those with active IBD and those in remission. Active IBD was defined on the basis of on clinical activity scoring, laboratory results, and endoscopic evaluation. The clinical characteristics of the patients, including age, sex, anthropometric measurements, disease duration, and location, were also recorded. The findings were compared between the groups with active IBD and in remission. Patients with other diseases that could affect the levels of s-LRG, CRP, erythrocyte sedimentation rate (ESR), and laboratory results, including extraintestinal complications, other autoimmune diseases, heart failure, infectious disease, and malignancy, were excluded. Signed informed consent forms were obtained from the participants and their parents before this study took place.

**Clinical activity:** The PCDAI was used to assess clinical disease activity in CD patients. Clinical symptoms were scored via the PUCAI in UC patients [3]. Clinical activation was defined as a PCDAI and PUCAI greater than 10 in CD patients and UC patients, respectively [11,12].

**Laboratory Testing:** Blood and urine samples were collected at the same time as those used for clinical scoring and endoscopic activity evaluations. Haemoglobin levels, platelet (PLT) counts, albumin, CRP, ESR, and LRG levels were analysed in the blood sample. Serum CRP (mg/L) and albumin (g/dL) levels were determined using immunoturbidimetry. The ESR was assessed according to the Westergren method (mm/h). The complete blood count of Hb and PLT was measured using a Hematology Analyzer. These parameters, which are part of a routine blood test, were all assessed using venous blood samples taken on the day the patients were admitted to hospital. In addition, the LRG levels were measured in the urine samples.

**Measurement of serum and urinary LRG levels**: Venous blood samples and urine samples were collected after 8–12 h of fasting. After centrifugation, these samples were portioned and stored at −20 °C until the analysis day. A commercial sandwich, the enzyme-linked immunosorbent assay (ELISA) kit (Elabscience Biotechnology Inc., Houston, TX, USA), was used to assess the LRG levels. The studies were performed according to the instructions in the kit package inserts. Spectrophotometric measurements were performed at a wavelength 450 nm via a Rayto (Microplate reader, RT-2100C, Wis-Medical, Shenzhen, China) model ELISA reader. The LRG concentrations of the samples were determined from the standard curve drawn via diluted standard absorbances. The results were expressed as µg/mL.

**Endoscopic activity:** Endoscopic disease activity was analysed in 78.5% of patients with active IBD who underwent a total colonoscopy. Colonoscopy could not be performed on other patients because they were unsuitable for anaesthesia or because the family refused. Disease activation was defined by clinical scoring and laboratory results in these patients. For CD patients, the endoscopic assessment was graded using the Simple Endoscopic Score for Crohn’s Disease (SES-CD) [8]. The Mayo endoscopic score (MES) was used in patients with UC [8]. Scoring was independently performed by two endoscopists who were blinded to the results of the serum and urine marker analyses and reviewed if the scores were different.

**Statistical analysis:** The power analysis was performed with the G*power 3.1 program, and the minimum sample size to be included was determined to be 40 in each group in the study. Categorical variables (sex, type of IBD, medications) were assessed via the χ2 test and were expressed as numbers and percentages. Kolmogorov–Smirnov and Shapiro–Wilk tests were performed to evaluate the normality of the continuous variables. Nonparametric continuous variables (median [minimum-maximum] age; z-score of weight, height, and BMI; duration of disease; PUCAI; PCDAI; haemoglobin; s-LRG; u-LRG; PLT count; albumin; CRP; and ESR) were analysed with the Mann–Whitney U test or Kruskal–Wallis test. The correlations between laboratory parameters and disease clinical and endoscopic activity scores were analysed via Spearman’s rank correlation coefficient. The relationships between disease clinical and endoscopic activity and laboratory parameters were analysed via logistic regression. The data were analysed with the Statistical Package for Social Sciences (SPSS) computer software (version 21.0; SPSS, Chicago, IL, USA) and MedCalc v12.5 software. A two-tailed *p*-value < 0.05 was considered significant.

**Ethical considerations:** An ethics committee approval was received from the ethical committee of the Tepecik Training and Research Hospital (2022/18-3).

## 3. Results

### 3.1. Characteristics of the Patients

A total of 84 patients, 42 (50%) patients with active IBD and 42 (50%) patients in remission, were included in this study. The characteristics, clinical data, and endoscopic activity scores of the patients are shown in Table 1.

### 3.2. Comparison of s-LRG, u-LRG, and Laboratory Parameters

The inflammatory indicators in laboratory parameters that PLT count, CRP, and ESR were significantly higher in patients with active IBD than in patients with disease remission (*p* = 0.000, *p* = 0.046, and *p* = 0.001, respectively). Albumin and Hb levels were compared between the two groups, and they were found to be lower in patients with active IBD (*p* = 0.041 and *p* = 0.009, respectively). Serum LRG concentrations were elevated in patients with active IBD compared with those in remission (*p* = 0.020). However, there were no difference in the u-LRG levels between the two groups (*p* = 0.407; Table 2).

**Figure 1:**
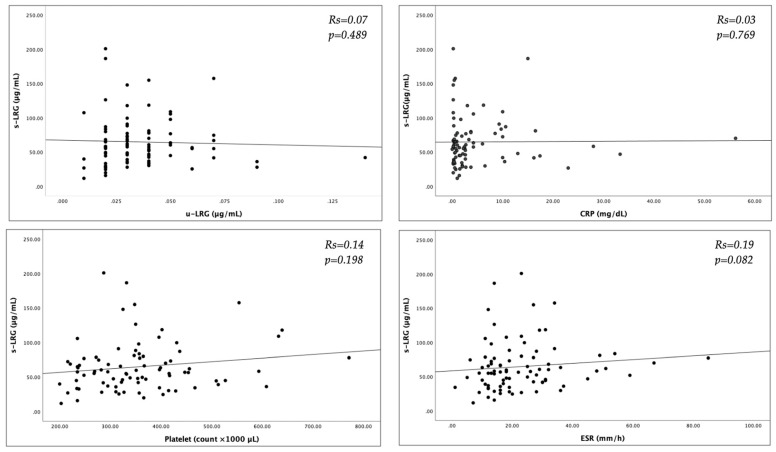
Correlations of serum leucine-rich alpha-2 glycoprotein 1 with various laboratory tests; s-LRG, serum leucine-rich alpha-2 glycoprotein 1, u-LRG, urine leucine-rich alpha-2 glycoprotein 1; CRP, C-reactive protein, ESR, erythrocyte sedimentation rate. Rs, Spearman’s rank correlation coefficient; the number of subjects was 84 per item.

In patients with IBD, positive correlations were observed between s-LRG, the PLT, CRP, and the ESR, but these correlations were not statistically significant (Figure 1). Thus, s-LRG was significantly negatively correlated with both albumin and Hb (*p* = 0.002, Rs = −0.26 and *p* = 0.014, Rs = −0.32, respectively). Urinary LRG was not correlated with s-LRG in any of the patients with IBD included or in patients with active IBD (*p* = 0.489 and *p* = 0.329, respectively). Although the u-LRG level was not correlated with the PLT, CRP level, ESR, or albumin level, a significant negative correlation was observed between the u-LRG level and the Hb level (*p* = 0.046).

### 3.3. Correlation of Serum LRG with Clinical and Endoscopic Activity Scoring

In patients with UC, according to the PUCAI, 50% (*n* = 31) achieved with clinic remission, 27.4% (*n* = 17) had mild clinical activation, 17.7% (*n* = 11) had moderate clinical activation, and 4.8% (*n* = 3) had severe clinical activation. According to the PCDAI in CD patients, 13 (59.1%) patients had clinical remission, 3 (13.6%) patients had mild clinical activation, 4 (18.2%) patients had moderate clinical activation, and 2 (9.1%) patients had severe clinical activation. The serum LRG was positively correlated with PUCAI and PCDAI, but it was not statistically significant (*p* = 0.055, Rs = 0.24 and *p* = 0.132, Rs = 0.33, respectively). Similarly, the MES and SES-CD were positively but not significantly correlated with s-LRG in patients with active IBD (*p* = 0.165, Rs = 0.28, and *p* = 0.779, Rs = 0.11, respectively).

The cutoff value for s- LRG (77.03 μg/mL) had a sensitivity and specificity of 40.4% (95% CI 25.6–56.7%) and 88.1% (95% CI 74.3–96.0%), respectively (Table 3).

### 3.4. Association Between Laboratory Parameters and Disease Activity in Patients with IBD

The relationship between laboratory parameters and disease activity in patients with IBD was assessed by means of logistic regression analysis. Disease activity was positively associated with s-LRG, ESR, CRP, and the PLT, whereas it was negatively associated with albumin and Hb (Table 4). It was found that s-LRG was a more significant parameter than CRP in predicting disease activation (*p* = 0.014 and *p* = 0.032, respectively). In addition, the PLT count was found to be the most significant parameter for predicting disease activation (*p* = 0.011).

## 4. Discussion

This prospective study showed that patients with active IBD had significantly elevated s-LRG levels compared to remission patients. Additionally, s-LRG levels were correlated with inflammatory indicators and clinical and endoscopic disease activity scores. However, the study revealed that u-LRG levels were not significantly different in patients with active IBD and were not correlated with s-LRG.

A study of adults revealed that s-LRG may be a biomarker reflecting IBD activity [8]. Shinzaki et al. [13] demonstrated that the levels of s-LRG were significantly increased and correlated with clinical and endoscopic activity in patients with UC. Komatsu et al. [14] showed that the LRG correlates with the IUS scores and proposed it as a new indicator for the targeting of transmural healing in adult patients with CD. Additionally, a recent study evaluating the effectiveness of s-LRG in demonstrating remission in children with IBD revealed that s-LRG levels were lower in patients who were in remission [15]. Similar to these studies, the present study showed that s-LRG levels were higher in patients with active IBD than in those in remission. In addition, this study showed that s-LRG has good specificity but low sensitivity for disease activation in patients with IBD. Yoshimura et al. [8] reported that s-LRG had a sensitivity of 55.6% and a specificity of 100%, with a similar cutoff value. While the results are similar, the practical cutoff value needs to be validated with large-scale studies.

Biomarkers and inflammatory indicators in laboratory parameters are useful predictors of recurrence and long-term prognosis and are minimally invasive in patients IBD. It is well known that inflammatory indicators of IBD for disease activation in laboratory parameters are anaemia, thrombocytosis, hypoalbuminemia, elevated CRP, and ESR [1]. The present study found lower levels of Hb and albumin, higher PLTs, and higher levels of CRP and ESR in patients with disease activation. In the literature, some studies have shown significant positive correlations with LRG for CRP, ESR, and PLT counts, whereas significant negative correlations have been found for albumin and Hb levels [8,15]. According to Takada et al. [16], LRG is a useful biomarker for predicting moderate to severe endoscopic activity in CD. In addition, assessing the combination LRG with CRP is useful, with high specificity when both are above the cut-off [16]. Karashima et al. [17], in a study that compared the LRG, CRP, and FC in the early phase after initiating induction therapy for active UC, showed that LRG at week 1 was a useful predictor of remission at week 8 as well as CRP, while FC was less accurate. In addition, they showed that LRG was correlated with excellent CRP. The s-LRG levels were correlated with inflammatory indicators in the laboratory parameters in the present study. The study also found that disease activation showed a positive association with s-LRG, ESR, CRP, and PLT count, whereas it was negatively associated with albumin and Hb. These results are similar to those of previous studies.

In patients with IBD, mucosal inflammation leads to the production of several cytokines such as IL-6, TNF-α, and IL-22. While serum CRP levels are mediated by IL-6, LRG production is not dependent on IL-6 alone [18]. In addition, the use of anti-TNFα antibody preparations and Janus kinase (JAK) inhibitors can result in a negative CRP. Therefore, it has been determined that s-LRG could reflect inflammation in patients with IBD better than CRP [19]. Previous studies have reported that patients with IBD have negative CRP results, even though they are endoscopically active [6,20]. A prospective study in adults showed that s-LRG was detectable in every patient, even when CRP was undetectable [8]. Additionally, LRG has been shown to be more effective than CRP as a marker for detecting endoscopic activity in patients with IBD and could be used for follow-up [4,21]. Similarly, it was found that s-LRG was a more significant parameter than CRP in predicting disease activation in the present study.

Yasuda et al. [15] reported positive correlations between s-LRG levels and the PUCAI and PCDAI for evaluating clinical remission. Shinzaki et al. [13] reported that serial measurements of LRG levels were significantly elevated in patients with an endoscopically active stage compared with those with a mucosal healing stage. It has been shown that there is a correlation between LRG levels and the MES in UC patients and as well as between LRG levels and the SES-CD score in CD patients [8]. In the present study, the endoscopic scores of patients—MES and SES-CD—were correlated with s-LRG levels in UC and CD patients with disease activation, respectively. Therefore, s-LRG may predict endoscopic activation in patients who are not suitable for endoscopic evaluation.

S-LRG and FC have been studied in adults with IBD. Both FC and s-LRG have been shown to have high specificity in predicting endoscopic remission [22]. However, biomarkers may be studied in the urine, which can be more easily obtained because of the difficulty of obtaining stool samples from children. Serum and urinary LRG have been researched for their diagnostic utility for paediatric acute appendicitis by Kakar et al. They reported that the diagnostic performance of s-LRG and u-LRG were non-invasive, rapid, and accurate biomarkers for acute appendicitis [23]. No study has investigated u-LRG in patients with IBD. This study evaluated the u-LRG and s-LRG levels in patients with IBD at the same time. However, u-LRG was not correlated with s-LRG or other inflammatory markers or the clinical and endoscopic activity scores of patients. This may be related to the fact that the u-LRG measurement is technically more difficult and requires more dilution. The mechanism of LRG excretion in urine is unclear. Previous reports have shown that LRG is increased in bacterial disease, expressed by neutrophils differentiating in the liver and high endothelial mesenteric venules such as the mesoappendix, and expressed at sites of inflammation [24,25]. Recently, u-LRG has been reported as a possible biomarker for immunoglobulin A nephropathy, steroid-resistant nephrotic syndrome, and diabetic kidney disease. Thus, u-LRG may reflect local inflammation, such as that in patients with appendicitis and renal injury. Therefore, the correlation between disease activation and urinary excretion of LRG may not have been demonstrated in patients with IBD where chronic and widespread inflammation is observed due to different causes and pathological mechanisms.

This study’s limitations include the fact that it was performed as a single-centre analysis and involved a limited number of participants. In addition, colonoscopies were performed in a small number of patients. For UC and CD patients, LRG could not be evaluated separately because of the insufficient distribution of the number of patients.

## 5. Conclusions

The serum LRG level is a useful biomarker for predicting disease activation in children with IBD and appears to be a more significant parameter than the CRP. However, the u-LRG level is not effective in predicting disease activation in children with IBD. Further large-scale studies are needed to determine the clinical benefits of using LRG as a biomarker for disease activation in children with IBD.

## Figures and Tables

**Table 1:** The characteristics, clinical, and endoscopic activity scoring of patients.

	Patients with Disease Activation (*n* = 42)	Patients in Remission (*n* = 42)	*p* Value
Age (*years*) (*Mean* ± *SD*)	14.09 ± 3.73	14.88 ± 2.38	0.709
Sex (*%* Female)	40.5	42.9	0.825
UC [*n* (*%*)]	32 (76.2)	30 (71.4)	0.804
CD [*n* (*%*)]	10 (23.8)	12 (28.6)	
PUCAI (*Median* (*min-max*))	32.7 (15–65)	0 (0–5)	0.000
PCDAI (*Median* (*min-max*))	37.5 (12.5–52.5)	0 (0–7.5)	0.000
Duration of disease (months) (*Mean* ± *SD*)	24.2 ± 19.5	29.4 ± 21.3	0.242
Weight (z-score) (*Median* (*min-max*))	−0.9 (−3.6–1.3)	−0.4 (−3.2–2.8)	0.690
Height (z-score) (*Median* (*min-max*))	−0.5 (−2.8–2.4)	−0.08 (−2.2–1.6)	0.016
BMI (z-score) (*Median* (*min-max*))	−0.6 (−5.2–1.8)	−0.32 (−4.9–2.3)	0.129
MES (*Mean* ± *SD*)	2.0 ± 0.6		
SES-CD (*Mean* ± *SD*)	6.6 ± 3.3		

UC, ulcerative colitis; CD, Crohn’s disease; min, minimum; max, maximum; BMI, body mass index; PUCAI, paediatric ulcerative colitis activity index; PCDAI, paediatric Crohn’s disease activity index; MES, Mayo endoscopic score, SES-CD, simple endoscopic score for Crohn’s disease.

**Table 2:** Laboratory parameters of patients.

Parameters	Patients with Disease Activation (*n* = 42)	Patients in Remission (*n* = 42)	*p* Value
Haemoglobin (g/dL) (*Mean* ± *SD*)	11.6 ± 2.3	12.7 ± 1.7	0.009
Platelet (count ×10^3^ μL) (*Mean* ± *SD*)	402 ± 123	310 ± 66	0.000
Albumin (g/dL) (*Mean* ± *SD*)	4.1 ± 0.6	4.4 ± 0.3	0.041
CRP (mg/dL) (*Median* (*min-max*))	2.6 (0.2–56.2)	1.2 (0.2–23.0)	0.046
ESR (mm/h) (*Mean* ± *SD*)	28.5 ± 17.0	17.4 ± 7.7	0.001
Serum LRG (μg/mL) (*Mean* ± *SD*)	75.2 ± 42.7	54.4 ± 25.4	0.020
Urinary LRG (μg/mL) (*Mean* ± *SD*)	0.03 ± 0.02	0.03 ± 0.01	0.407

CRP, C-reactive protein; min, minimum; max, maximum; ESR, erythrocyte sedimentation rate; LRG, leucine-rich alpha-2 glycoprotein 1.

**Table 3:** The diagnostic value of the serum LRG for the prediction of disease activation in patients with IBD.

	Sensitivity, %	Specificity, %	PPV, %	NPV, %
Serum LRG	40.4 (25.6–56.7)	88.1 (74.3–96.0)	77.2 (58.0–89.3)	59.6 (52.9–66.0)

LRG, leucine-rich alpha-2 glycoprotein 1; PPV, positive predictive value; NPV, negative, predictive value.

**Table 4:** Logistic regression of laboratory parameters of patients with disease activation.

Parameter	Univariate Regression Model		Multivariate Regression Model	
	OR (95% CI Lower–Upper)	*p* Value	OR (95% CI Lower–Upper)	*p* Value
Serum LRG (μg/mL)	0.981 (0.967–0.996)	0.014	0.981 (0.967–0.996)	0.044
CRP (mg/dL)	0.903 (0.823–0.991)	0.032		
ESR (mm/h)	0.923 (0.879–0.969)	0.001	0.948 (0.903–0.996)	0.036
Albumin (g/dL)	3.963 (1.308–12.007)	0.015		
Haemoglobin (g/dL)	1.330 (1.056–1.674)	0.015		
Platelet (count ×10^3^ μL)	0.989 (0.983–0.995)	0.001	0.991 (0.985–0.998)	0.011

LRG, leucine-rich alpha-2 glycoprotein 1; CRP, C-reactive protein; ESR, erythrocyte sedimentation rate.

## Data Availability

The datasets supporting the conclusions of this article are included within the article.

## Abbreviations

The following abbreviations are used in this manuscript:

IBD	Inflammatory bowel disease
CD	Crohn’s disease
UC	Ulcerative colitis
IBD-U	Unclassified inflammatory bowel disease
PCDAI	Paediatric Crohn’s Disease Activity Index
PUCAI	Paediatric Ulcerative Colitis Activity Index
CRP	C-reactive protein
FC	Faecal calprotectin
LRG	Leucine rich α-2 glycoprotein
IL	Interleukin
TNF	Tumour necrosis factor
s-LRG	Serum leucine rich α-2 glycoprotein
u-LRG	Urinary leucine rich α-2 glycoprotein
ESR	Erythrocyte sedimentation rate
PLT	Platelet
ELISA	Enzyme-linked immunosorbent assay
SES-CD	Simple endoscopic score for Crohn’s disease
MES	Mayo endoscopic score
SPSS	Statistical Package for Social Sciences
